# Metabolic status indicators and influencing factors in non-obese, non-centrally obese nonalcoholic fatty liver disease

**DOI:** 10.1097/MD.0000000000032922

**Published:** 2023-02-10

**Authors:** Zhipeng Huang, Donghong Wei, Xueping Yu, Zicheng Huang, Yijie Lin, Wenji Lin, Zhijun Su, Jianjia Jiang

**Affiliations:** a Department of Gastroenterology, First Hospital of Quanzhou Affiliated to Fujian Medical University, Quanzhou, Fujian, China; b Department of Preventive Medicine, School of Inspection and Prevention, Quanzhou Medical College, Quanzhou, Fujian, China; c Department of Infectious Diseases, First Hospital of Quanzhou Affiliated to Fujian Medical University, Quanzhou, Fujian, China; d Department of Radiology, First Hospital of Quanzhou Affiliated to Fujian Medical University, Quanzhou, Fujian, China; e Department of Endocrinology, First Hospital of Quanzhou Affiliated to Fujian Medical University, Quanzhou, Fujian, China.

**Keywords:** insulin resistance, metabolic syndrome, nonalcoholic fatty liver disease, obesity, visceral adiposity

## Abstract

Non-obese nonalcoholic fatty liver disease (NAFLD) is characterized by metabolic disorders and related complications. This study aimed to provide an integrated description of clinical, metabolic, and influencing factors for a specific category of patients with non-obese NAFLD. A total of 36 participants with body mass index (BMI) < 28 kg/m^2^ and visceral adipose tissue < 100 cm^2^ were classified into 2 groups: the non-obese, non-centrally obese control group (n = 17) and non-obese, non-centrally obese NAFLD group (n = 19). Hypertriglyceridemia, impaired fasting glucose, low high-density lipoprotein cholesterol levels, and hypertension were used to determine whether participants were metabolically abnormal. Based on a logistic regression model, odds ratios for the factors influencing NAFLD with 95% confidence intervals were calculated. Insulin resistance (IR) and fasting plasma glucose (FPG) levels were higher in the NAFLD group than in the control group (*P* < .05). The NAFLD group had a higher metabolic abnormality rate than the healthy control group (36.84% vs 5.88%, *P* = .044). Correlation analysis showed that IR was positively correlated with FPG and triglyceride (*P* < .05). BMI was the main influencing factor of NAFLD (regression coefficient *β* = 0.631; odds ratio = 1.879; 95% confidence interval, 1.233–2.863). NAFLD patients with a BMI < 28 kg/m^2^ and visceral adipose tissue < 100 cm^2^ had more apparent IR, higher FPG, and a higher metabolic abnormality rate. IR may be affected by FPG and triglyceride. Even in non-obese and non-centrally obese individuals, BMI should be controlled to avoid NAFLD.

## 1. Introduction

Nonalcoholic fatty liver disease (NAFLD) is considered a disease of affluent society since its association with metabolic syndrome, obesity, diabetes mellitus, dyslipidemia, and hypertension.^[1]^ Epidemiology studies suggest that the prevalence of NAFLD in Asia is comparable to that of Western countries, and is related to the “junk food” diet.^[2]^ Its clinical manifestations are not confined to the liver and are associated with a higher likelihood of cardiovascular disease (CVD), which causes heavy health and social burdens.^[3]^ Epidemiological evidence suggests that not all obese people develop NAFLD, and non-obese adults of all races may develop NAFLD.^[4]^

A previous study reported that approximately 8 to 19% of Asians with a body mass index (BMI) < 25 kg/m^2^ suffered from NAFLD, steatohepatitis, and fibrosis.^[5]^ In other studies on non-obese NAFLD, BMI was used to distinguish obesity; however, the criteria were different in the West and East.^[6]^ Most Asian studies have used BMI > 25 kg/m^2^ as the obesity threshold,^[7,8]^ whereas the standard of obesity in China is BMI > 28 kg/m^2^.^[9]^ However, because participants with similar BMI may have different levels of visceral fat accumulation, it is necessary to use a method that can accurately reflect the distribution of body fat tissue.

BMI reflects general obesity, and previous studies on non-obese NAFLD may have missed some individuals with abdominal obesity, owing to distinguishing obesity by BMI alone. Waist circumference (WC) is commonly used as a measure of abdominal obesity in clinical practice and can better reflect visceral or abdominal fat storage status to compensate for BMI deficiency to a certain extent.^[10]^ However, WC includes subcutaneous and intra-abdominal fat, which cannot fully represent visceral fat. In addition, there is a view that subcutaneous fat may play a role in storing excess fat and may protect participants from NAFLD damage by storing excess fat.^[11]^

Visceral adipose tissue (VAT) is regarded as the precise standard for visceral obesity; a person with a VAT < 100 cm^2^ is believed to have no excess visceral fat accumulation.^[12]^ Using VAT instead of WC eliminates the effects of subcutaneous adipose tissue (SAT). However, BMI is seldom combined with VAT in studies of non-obese, non-centrally obese NAFLD patients. Therefore, our research proposed that BMI < 28 kg/m^2^ combined with VAT < 100 cm^2^ should be regarded as a segmentation point for non-obesity. Simultaneously, the influence of general and abdominal obesity was eliminated and non-obesity was redefined. Based on this, the influencing factors and metabolic status of non-obese and non-centrally obese populations were studied further.

Increasing attention has been paid to studies on insulin resistance (IR) and the metabolic status of non-obese NAFLD patients; however, there is a lack of official guidelines or expert consensus, and the role of IR in non-obese, non-centrally obese NAFLD has not been clearly explained.^[13,14]^ Especially for the NAFLD category studied, visceral obesity, general obesity, IR, and related metabolic abnormalities were typically not present. However, whether there is a correlation between IR and metabolic indices remains unclear.

## 2. Patients and Methods

### 2.1. Study population

This was a cross-sectional study, and relatively little data have been documented for these specific populations. Between August 2020 and August 2022, participants who presented at our hospital underwent liver ultrasonography and provided written informed consent for VAT were included. Inclusion criteria were as follows: age > 18 years and clinically suspected diagnosis of NAFLD (including ultrasonography). The exclusion criteria were as follows: excessive alcohol intake: men drink >20 g per day and women drink >10 g a day; Hepatitis B and C; drug-induced hepatitis; autoimmune liver disease, including primary sclerosing cholangitis, immune hepatitis, and primary biliary cholangitis; and metabolic liver diseases, including glycogen accumulation disease, hemochromatosis, Wilson disease, α-1 antitrypsin deficiency, and cholesterol storage disorders.^[15]^ As the region is an advanced coastal city, the participants were from a combination of urban and rural areas. The diet of the patient cohort not only retains Chinese characteristics but is also affected by Western food to varying degrees. A total of 163 participants underwent VAT examination; 92 participants with a BMI ≥ 28 kg/m^2^ and 35 with a VAT ≥ 100 cm^2^ were excluded. A total of 36 participants with BMI < 28 kg/m^2^ and VAT < 100 cm^2^ were included. The participants were finally categorized into 2 groups: the non-obese, non-centrally obese NAFLD group (n = 19) and the non-obese, non-centrally obese, and non-NAFLD healthy control group (n = 17).

The Ethics Committee of First Hospital of Quanzhou Affiliated to Fujian Medical University approved the study (decision no. 2020-183).

### 2.2. Definition of non-obese and non-centrally obese

Since the Japanese Examination Committee for “Obesity Disease” proposed VAT ≥ 100 cm^2^ as high and VAT < 100 cm^2^ as normal in 2002,^[16]^ this standard has been widely adopted in various studies on abdominal obesity.^[12,17,18]^ In this study, a VAT ≥ 100 cm^2^ was defined as visceral obesity. Although the World Health Organization suggests that the BMI cutoff for obesity in Asian people should be 27.5 kg/m^2,[19]^ research on Asian NAFLD has used lower BMI thresholds for obesity (BMI ≥ 25 kg/m^2^).^[20]^ However, the definition of obesity by Chinese public health authorities has been consistent with a BMI ≥ 28 kg/m^2^ for several years.^[9,21,22]^ In this study, BMI + VAT was used to define obesity; BMI < 28 kg/m^2^ and VAT < 100 cm^2^ were strictly defined as non-obese.

### 2.3. Definition of abnormal metabolism

Four metabolic factors, blood glucose, hypertriglyceridemia, low high-density lipoprotein cholesterol (HDL-C), and hypertension, were used to determine whether the participants had normal metabolism. An impaired fasting blood glucose level was defined as fasting plasma glucose (FPG) ≥ 5.6 mmol or medical treatment for hyperlipidemia. Systolic pressure ≥ 130 mm Hg and/or diastolic pressure ≥ 85 mm Hg or hypertension treatment was used to describe hypertension. A hypertriglyceridemia patient was defined as having triglyceride (TG) ≥ 1.7 mmol/L or being on drug treatment for hyperlipidemia. Low HDL-C was regarded as HDL-C < 1.03 mmol/L in males or HDL-C < 1.29 mmol/L in females.^[23]^ If there were 1 or 0 abnormal metabolic indices, metabolism was considered normal, and >2 abnormal metabolic indices were considered abnormal metabolism.^[24,25]^

### 2.4. Measurements

Blood samples were collected after fasting for >8 hours. FPG, aspartate transaminase, gamma-glutamyl transferase (GGT), low-density lipoprotein cholesterol, alanine transaminase, total cholesterol, alkaline phosphatase, HDL-C, TG, and creatinine levels were measured using the automated biochemical analyzer AU5821 (Beckman, Brea, CA). Microparticle chemiluminescence was used as the determination method, and the original Beckman reagent and calibrator were used for the FPG determination. A DXI800 automatic chemiluminescence analyzer (Beckman , Brea, CA) was used to determine fasting insulin (FINS) levels, and the test kit included the original Beckman reagent and calibrator. Based on the homeostasis model assessment of IR (HOMA-IR), IR was estimated as HOMA-IR = FINS (IU/mL) × FPG (mmol/L)/22.5.

Height, weight, WC, BMI, and waist-to-height ratio (WHtR) = WC/height were determined and calculated according to standard methods and references.^[26]^ In accordance with a standard protocol, an intelligent pressurized electronic sphygmomanometer was used to measure diastolic and systolic blood pressure (DBP, SBP) (model HEM-7211, OMRON, Kyoto, Japan), and the final results were calculated by averaging 3 measurements.

### 2.5. Liver ultrasound measures and VAT measures

Sonographic examinations were performed by senior sonographers to compensate for observer bias. VAT measurements using computed tomography (CT) were performed by a competent professional radiologist. Liver ultrasonography was performed using an EPIQ5 color Doppler ultrasound system (Philips Company, Amsterdam, Netherlands). Ultrasound evaluation was performed according to the guidelines for prevention and treatment of NAFLD by the Chinese Society of Hepatology in 2018, and an enhanced near-field echo (“bright liver”), attenuated far-field echo, and unclear intrahepatic ductal structure were used to diagnose fatty liver.^[27]^ VAT and SAT were detected by abdominal scanning using LightSpeed VCT (GE Healthcare, Chicago, IL). The participants were scanned in the supine position at the umbilical level with a slice thickness of 5 mm.^[28]^ Self-equipped CT software was used to analyze the image; connected voxels with a CT attenuation range of −150 to −50 Hounsfield units were identified as fat, and VAT and SAT were calculated.^[29]^

### 2.6. Statistical analysis

R software, version 4.1.0 (R Foundation for Statistical Computing, Vienna, Austria) was used for the statistical analysis. Data with a normal distribution are summarized as mean ± standard deviation (x̅ ± s), whereas variables with a non-normal distribution are represented as median (interquartile range) [*M (P*_25_–*P*_75_)]. For normally distributed variables, comparisons between groups were performed using the independent samples *t* test. For non-normally distributed variables, the Mann–Whitney *U* test was used. Differences between categorical data were compared using the chi-square test. Correlation relationships were described using Pearson or Spearman correlation coefficient. A heat map was drawn using R software (version 4.1.0) to describe the correlation. Risk factor analysis was based on logistic regression analysis (backward method); the inclusion criterion was 0.05, whereas the elimination criterion was 0.10.

## 3. Results

Clinical and laboratory characteristics of participants.

### 3.1. Anthropometric, biochemical, and metabolic data

In total, 36 participants were included in the study. The clinical and laboratory parameters of the participants with (n = 19) and without (n = 17) NAFLD are shown in Table 1. The average age of NAFLD group was 37.22 ± 8.37 years, with 52.63% males; that of the control group was 33.86 ± 8.73 years, with 23.53% males. Sex and age were not statistically significant (*P* > .05). The NAFLD group had significantly higher BMI, WC, WHtR, VAT, FPG, FINS, IR, and GGT levels (*P* < .05) (Table 1). The differences between the 2 groups were not significant for SBP, DBP, SAT, alanine transaminase, aspartate transaminase, alkaline phosphatase, total cholesterol, low-density lipoprotein cholesterol, HDL-C, TG, or creatinine (*P* > .05) (Table 1).

**Table 1:** Anthropometrical and biochemical parameters in the non-obese NAFLD and control groups.

Variables	The control group N = 17	NAFLD group N = 19	*χ*²/*t*/*Z*	*P* values
Males	4	10	3.197	.097
Age	33.86 ± 8.73	37.22 ± 8.37	1.180	.246
BMI (kg/m^2^)	22.68 ± 2.41	25.58 ± 1.74	4.177	.000
WC (cm)	81.29 ± 7.09	89.56 ± 8.54	3.140	.003
WHtR	0.480 (0.45, 0.53)	0.52 (0.46, 0.55)	87.00	.018
SBP (mm Hg)	122.50 (110.00, 130.00)	120.07 (115.00, 132.00)	158.00	.910
DBP (mm Hg)	72.50 (60, 80)	70 (69.22, 70.50)	154.00	.810
VAT (cm^2^)	51.05 ± 22.46	71.09 ± 22.67	2.660	.012
SAT (cm^2^)	168.11 ± 86.44	213.56 ± 70.46	1.737	.091
ALT (U/L)	18.50 (13.5, 23.00)	22.5 (19.5, 39.3)	103.00	.063
AST (U/L)	21.78 ± 6.97	24.52 ± 16.59	0.632	.532
ALP (U/L)	62.80 ± 17.43	53.99 ± 28.92	−1.033	.310
GGT (U/L)	18.00 ± 7.77	34.89 ± 23.96	2.828	.010
TC (mmol/L)	5.01 ± 1.21	4.33 ± 1.46	−1.520	.138
LDL-C (mmol/L)	3.28 ± 0.99	2.81 ± 1.14	−1.299	.203
HDL-C (mmol/L)	1.30 (1.10, 1.76)	1.25 (1.09, 2.05)	154.00	.812
TG (mmol/L)	0.99 (0.62, 1.44)	1.23 (0.67, 2.58)	120.50	.194
FPG (mmol/L)	4.94 (4.75, 5.18)	5.81 (5.12, 6.29)	79.00	.009
INS (mU/L)	7.27 ± 2.93	10.40 ± 5.47	2.100	.043
HOMA-IR	1.69 ± 0.72	2.71 ± 1.21	3.038	.005
CRE (μmol/L)	66.45 ± 19.68	60.76 ± 28.20	−0.694	.492
URIC (mol/L)	247.00 (192.75, 398.25)	327.50 (215.75, 385.25)	132	.350

ALP = alkaline phosphatase, ALT = alanine transaminase, AST = aspartate transaminase, BMI = body mass index, CRE = creatinine, DBP = diastolic blood pressure, FPG = fasting plasma glucose, GGT = gamma-glutamyltransferase, HDL-C = high-density lipoprotein cholesterol, HOMA-IR = homeostasis model assessment of insulin resistance, INS = insulin, LDL-C = low-density lipoprotein cholesterol, NAFLD = nonalcoholic fatty liver disease, SAT = subcutaneous adipose tissue, SBP = systolic blood pressure, TC = total cholesterol, TG = triglyceride, URIC = uric acid, VAT = visceral adipose tissue, WC = waist circumference, WHtR = waist-to-height ratio.

### 3.2. Comparison of metabolic abnormality rate

The indicators FPG, TG, SBP, DBP, and HDL-C were used to define metabolic state. According to these criteria, at least 2 abnormal metabolic indicators must exist to be classified as metabolic abnormalities. The metabolic abnormality rate in the NAFLD group was higher than that in the control group (36.84% vs 5.88%, *P* = .044, Table 2).

**Table 2:** The metabolic abnormality rate in non-obese NAFLD and control groups.

	Metabolic abnormalities		Total	*χ*²	*P* values
	No	Yes			
The control group	16 (94.12%)	1 (5.88%)	17	–	
NAFLD group	12(63.16%)	7 (36.84%)	19		.044
Total	28 (77.78%)	8 (22.22%)	36		

NAFLD = nonalcoholic fatty liver disease.

### 3.3. Correlation analysis

**Figure 1.**
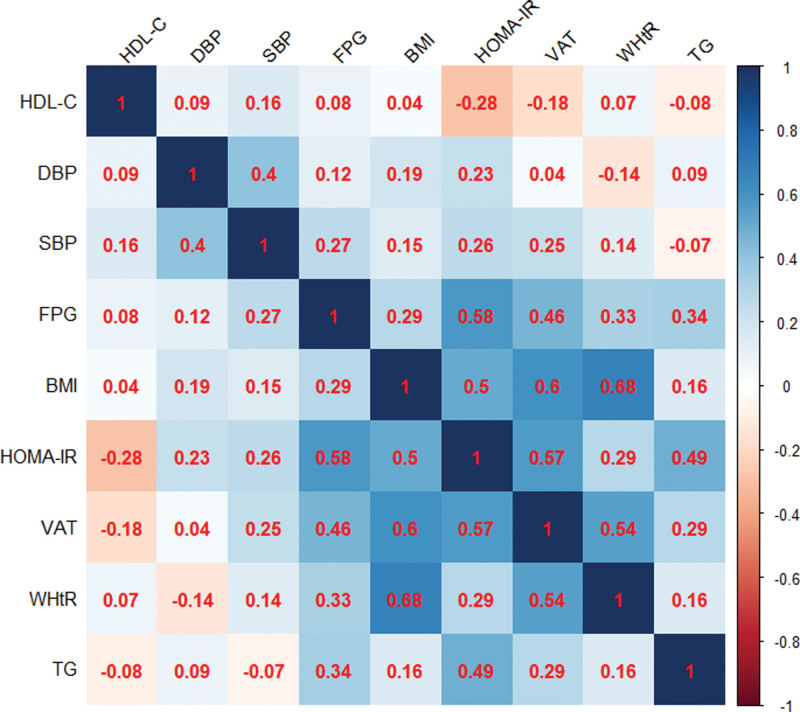
Correlation analysis of indicators used to define metabolic status. Blue represents a positive correlation between the 2 variables and red represents a negative correlation between them. The darker the color, the higher the correlation; the lighter the color, the lower the correlation. BMI = body mass index, DBP = diastolic blood pressure, FPG = fasting plasma glucose, HDL-C = high-density lipoprotein cholesterol, HOMA-IR = homeostasis model assessment of insulin resistance, SBP = systolic blood pressure, TG = triglyceride, VAT = visceral adipose tissue, WHtR = waist-to-height ratio.

The indicators FPG, TG, SBP, DBP, and HDL-C were used to define metabolic state. As illustrated in Figure 1, FPG and TG levels were positively correlated with IR (*P* < .05), whereas SBP, DBP, and HDL-C levels were not correlated with IR (*P* > .05). SBP, DBP, FPG, TG, and HDL-C levels did not correlate with BMI (*P* > .05) or WHtR (*P* > .05). SBP, DBP, HDL-C, and TG were not correlated with VAT (*P* > .05), whereas FPG was positively correlated with VAT (*P* < .05).

### 3.4. A backward Wald binary logistic regression analysis of NAFLD-related factors

BMI, WC, WHtR, VAT, FPG, HOMA-IR, FINS, and GGT were significant indicators in univariate analysis, and it was also performed a logistic regression analysis. BMI was an influencing factor for non-obese and non-centrally obese patients with NAFLD with BMI < 28 kg/m^2^ and VAT < 100 cm^2^ (regression coefficient *β* = 0.631; odds ratio = 1.879; 95% confidence interval, 1.233–2.863).

## 4. Discussion

In this study, even when BMI was < 28 kg/m^2^ and VAT was < 100 cm^2^, the metabolic abnormality rate of NAFLD was 36.84%. Patients with NAFLD had higher IR and FPG levels in these populations. After adjustment for possible confounders, BMI remained the most important influencing factor for NAFLD.

In studies of NAFLD, if obesity is simply defined by BMI, it is easy to exclude some individuals with abdominal obesity. Based on previous research on normal-weight non-central obesity-NAFLD, we opened a new perspective.^[30]^ However, the authors of that study set BMI < 25 kg/m^2^ as the obesity cutoff point and WC as the evaluation index of abdominal obesity. Chinese individuals require stricter definitions of abdominal obesity.^[31]^ We further propose considering BMI < 28 kg/m^2^ and VAT < 100 cm^2^ as the cutoff points for non-obesity, thereby eliminating the effects of overall and abdominal obesity and strictly limiting the conditions of non-obesity. This should help to distinguish the specific NAFLD population and understand their metabolic status indicators and influencing factors.

Recently, the metabolic status of non-obese individuals has received increased attention. Excess adipose tissue can release many lipid metabolites and proinflammatory cytokines and mediate IR. Non-obese NAFLD lacks obvious extra adipose tissue and its pathogenesis and clinical outcomes may differ. However, studies have shown that non-obese and obese patients with NAFLD have common alterations in metabolism and the cardiovascular system.^[32,33]^ Compared with overweight and obese NAFLD, lean NAFLD also has full-spectrum characteristics of metabolic syndrome risk factors, such as higher blood glucose levels, blood lipid levels, SBP, DBP, and WC.^[34]^ This was similar to our study, in which FPG and IR levels were higher in the NAFLD group (*P* < .05). However, our study did not show the comprehensive features of metabolic abnormalities after strictly limiting the definition of obesity. In our study, HDL-C, FPG, SBP, TG, and DBP were used to define the metabolic state; however, TG, SBP, DBP, and HDL-C levels were not significantly different between the 2 groups (*P* > .05). IR is often considered a key element in the development of NAFLD and is closely associated with NAFLD, CVD, and metabolic disorders. Therefore, we attempted to identify an association between IR and metabolic indicators. In our study, SBP, DBP, FPG, TG, and HDL-C, which were used to define metabolic status, were not correlated with BMI; however, FPG and TG levels were positively correlated with IR. Dyslipidemia and hyperglycemia are considered the most crucial contributors to CVD events, and insulin sensitizers are commonly used to ameliorate these metabolic abnormalities.^[35]^ Evidence suggests a parallel increase in the incidences of NAFLD, CVD, IR, and metabolic disorders. Thus, CVD and NAFLD/nonalcoholic steatohepatitis (NASH) may be associated with type 2 diabetes mellitus.^[36]^ IR may be affected by FPG and TG levels in non-obese, non-centrally obese patients with NAFLD.

People of normal weight with metabolic disorders have a higher risk of developing NAFLD than those who are metabolically healthy.^[13]^ A recent multicenter study in Asia showed that coexisting metabolic comorbidities were prevalent in non-obese individuals with NAFLD, including diabetes or prediabetes (71.1%), dyslipidemia (70.1%), and hypertension (43.4%).^[37]^ In our study, the only difference was that, in comparison with metabolic parameters alone, only FPG was statistically different between the 2 groups. However, when >2 abnormal metabolic indices were used as the metabolic abnormality standards, the metabolic abnormality rate in the NAFLD group was 36.84%, which was higher than that in the healthy control group. Another relevant difference is that other studies described baseline characteristics using BMI and WC to define obesity. There are few studies on the metabolic abnormality rate in this specific NAFLD population as defined by the metabolic abnormality criteria, which is also a contribution of this study.

Whether metabolic abnormalities are the common underpinning of NAFLD, independent of abdominal obesity and overall obesity, remains unclear. To date, little is known about the influencing factors of this part of the population, which is also one of the highlights of this study and is worthy of further exploration. In a recent study of non-obese people undergoing physical examination, TG levels best predicted non-obese fatty liver disease.^[38]^ However, that study focused on non-obese participants, whereas we focused on the joint definition of non-centrally obese and non-obese participants. Another difference was that they defined non-obesity as BMI < 24 kg/m^2^. Even when the joint diagnostic criteria were used to strictly limit the definition of obesity, we found no significant difference in lipid levels between patients with NAFLD and control participants. In a famous Framingham Heart Study, VAT and TG were found to be closely related in both men and women.^[39]^ In our study, the VAT of all participants was within the normal range, which may explain why there was no significant difference in the TG levels.

In our study, compared with the control participants, the NAFLD group with BMI < 28 kg/m^2^ and VAT < 100 cm^2^ had higher BMI, WC, WHtR, VAT, IR, and GGT levels and more apparent metabolic disorders. Logistic regression analysis revealed that BMI was the main factor influencing NAFLD. In a study of healthy adults who had donated a liver, as assessed by liver biopsy, NAFLD/NASH was found to be common even in healthy lean individuals, and higher serum uric acid levels and BMI were the 2 main risks of NAFLD/NASH in these individuals. Similar to our study, they defined non-obese and lean participants as those with a BMI < 30 kg/m^2^ and <25 kg/m^2^, respectively.^[40]^ Non-obese and non-centrally obese individuals with a BMI < 28 kg/m^2^ and VAT < 100 cm^2^ are not completely healthy, and there is a possibility of reducing body fat. For patients with BMI < 28 kg/m^2^ and VAT < 100 cm^2^, in addition to reducing fat, a reasonable diet structure, improving lifestyle, controlling other metabolic risk factors, alleviating IR, inhibiting persistent low-grade inflammation, maintaining intestinal microecological balance should be considered to delay the progression of liver disease and prevent extrahepatic disease. Strong evidence in recent years shows that even when non-obesity is defined by a BMI < 25 kg/m^2^, lifestyle interventions and moderate weight loss are meaningful for the remission of NAFLD in non-obese patients.^[41]^ In other words, to change NAFLD status, a lower BMI should be pursued more strictly, even in patients without central obesity.

This study had some limitations. Relatively few patients with non-obese and non-centrally obese NAFLD met the conditions of this study, partly because the coronavirus disease 2019 pandemic affected data collection. However, a major highlight of this study was the strictly limited definition of non-obese and non-centrally obese NAFLD. The diagnosis of NAFLD was not supported by pathological or nuclear magnetic resonance evidence, which is either invasive or expensive, and we were able to compensate for this observer bias with an experienced ultrasonic examination expert.

In conclusion, according to the findings of this study, patients with NAFLD with BMI < 28 kg/m^2^ and VAT < 100 cm^2^ had more apparent IR, higher FPG, and a higher metabolic abnormality rate. IR may be affected by the FPG and TG levels. Even in non-obese and non-centrally obese individuals, BMI should be controlled to avoid NAFLD.

## Acknowledgments

The authors appreciate the Gastroenterology Department of Fudan Zhongshan Hospital for the support rendered.

## Author contributions

**Conceptualization:** Zhipeng Huang, Donghong Wei, Xueping Yu, Jianjia Jiang.

**Data curation:** Zhipeng Huang, Donghong Wei.

**Formal analysis:** Zhipeng Huang, Donghong Wei.

**Funding acquisition:** Zhipeng Huang.

**Investigation:** Zhipeng Huang, Zicheng Huang, Yijie Lin, Jianjia Jiang.

**Methodology:** Zhipeng Huang, Yijie Lin, Wenji Lin, Jianjia Jiang.

**Project administration:** Zhipeng Huang, Zicheng Huang, Zhijun Su, Jianjia Jiang.

**Resources:** Zhipeng Huang, Jianjia Jiang.

**Software:** Zhipeng Huang, Donghong Wei.

**Supervision:** Zhipeng Huang, Zhijun Su, Jianjia Jiang.

**Validation:** Zhipeng Huang, Wenji Lin, Jianjia Jiang.

**Visualization:** Zhipeng Huang, Donghong Wei, Xueping Yu.

**Writing – original draft:** Zhipeng Huang, Donghong Wei, Xueping Yu.

**Writing – review & editing:** Zhipeng Huang, Donghong Wei, Xueping Yu.

## References

[1] Abenavoli L Di Renzo L Boccuto L. Health benefits of Mediterranean diet in nonalcoholic fatty liver disease. Expert Rev Gastroenterol Hepatol. 2018;12:873–81. 30033779 10.1080/17474124.2018.1503947

[2] Guo W Ge X Lu J. Diet and risk of non-alcoholic fatty liver disease, cirrhosis, and liver cancer: a large prospective cohort study in UK Biobank. Nutrients. 2022;14:5335. 36558494 10.3390/nu14245335 PMC9788291

[3] Kim SH Park HY Lee HS. Association between non-alcoholic fatty liver disease and coronary calcification depending on sex and obesity. Sci Rep. 2020;10:1–8. 31974458 10.1038/s41598-020-57894-y PMC6978333

[4] Kim JJ Kim D Yim JY. Polycystic ovary syndrome with hyperandrogenism as a risk factor for non-obese non-alcoholic fatty liver disease. Aliment Pharmacol Ther. 2017;45:1403–12. 28370150 10.1111/apt.14058

[5] Fan JG Kim SU Wong VW. New trends on obesity and NAFLD in Asia. J Hepatol. 2017;67:862–73. 28642059 10.1016/j.jhep.2017.06.003

[6] Lee G You HJ Bajaj JS. Distinct signatures of gut microbiome and metabolites associated with significant fibrosis in non-obese NAFLD. Nat Commun. 2020;11:4982. 33020474 10.1038/s41467-020-18754-5 PMC7536225

[7] Lee SW Lee TY Yang SS. Risk factors and metabolic abnormality of patients with non-alcoholic fatty liver disease: either non-obese or obese Chinese population. Hepatob Pancreat Dis. 2018;17:45–8. 10.1016/j.hbpd.2018.01.007 29428103

[8] Tobari M Hashimoto E Taniai M. Characteristics of non-alcoholic steatohepatitis among lean patients in Japan: not uncommon and not always benign. J Gastroen Hepatol. 2019;34:1404–10. 10.1111/jgh.14585 30590868

[9] Tao J Wang Y Li L. Critical roles of ELVOL4 and IL-33 in the progression of obesity-related cardiomyopathy via integrated bioinformatics analysis. Front Physiol. 2020;11:542. 32581837 10.3389/fphys.2020.00542 PMC7291781

[10] Liu HK Yang MC Su YT. Novel ultrasonographic fatty liver indicator can predict hepatitis in children with non-alcoholic fatty liver disease. Front Pediatr. 2019;6:416. 30671426 10.3389/fped.2018.00416 PMC6331417

[11] Huang JF Tsai PC Yeh ML. Risk stratification of non-alcoholic fatty liver disease across body mass index in a community basis. J Formos Med Assoc. 2020;119:89–96. 30952479 10.1016/j.jfma.2019.03.014

[12] Kong M Xu M Zhou Y. Assessing visceral obesity and abdominal adipose tissue distribution in healthy populations based on computed tomography: a large multicenter cross-sectional study. Front Nutr. 2022;9:871697. 35548570 10.3389/fnut.2022.871697 PMC9082940

[13] Vusirikala A Thomas T Bhala N. Impact of obesity and metabolic health status in the development of non-alcoholic fatty liver disease (NAFLD): a United Kingdom population-based cohort study using the health improvement network (THIN). BMC Endocr Disord. 2020;20:96. 32605642 10.1186/s12902-020-00582-9 PMC7325099

[14] Eslam M Fan JG Mendez-Sanchez N. Non-alcoholic fatty liver disease in non-obese individuals: the impact of metabolic health. Lancet Gastroenterol. 2020;5:713–5. 10.1016/S2468-1253(20)30090-X 32413341

[15] Wong VW Chan WK Chitturi S. Asia-Pacific working party on non-alcoholic fatty liver disease guidelines 2017-part 1: definition, risk factors and assessment. J Gastroenterol Hepatol. 2018;33:70–85. 28670712 10.1111/jgh.13857

[16] Examination Committee of Criteria for ‘Obesity Disease’ in Japan; Japan Society for the Study of Obesity. New criteria for “obesity disease” in Japan. Circ J. 2002;66:987–92. 12419927 10.1253/circj.66.987

[17] Yan H Qin Q Chen J. Gut microbiome alterations in patients with visceral obesity based on quantitative computed tomography. Front Cell Infect Microbiol. 2021;11:823262. 35127566 10.3389/fcimb.2021.823262 PMC8811355

[18] Ohashi S Maruno T Fukuyama K. Visceral fat obesity is the key risk factor for the development of reflux erosive esophagitis in 40-69-years subjects. Esophagus. 2021;18:889–99. 34117973 10.1007/s10388-021-00859-5 PMC8387261

[19] Jang K Tong T Lee J. Altered gene expression profiles in peripheral blood mononuclear cells in obese subjects. Obes Facts. 2020;13:375–85. 32544907 10.1159/000507817 PMC7445570

[20] Ye J Wu Y Li F. Effect of orlistat on liver fat content in patients with nonalcoholic fatty liver disease with obesity: assessment using magnetic resonance imaging-derived proton density fat fraction. Therap Adv Gastroenterol. 2019;12:1756284819879047. 10.1177/1756284819879047 PMC6767749 31632457

[21] Bae JC Cho NH Kim JH. Association of body mass index with the risk of incident type 2 diabetes, cardiovascular disease, and all-cause mortality: a community-based prospective study. Endocrinol Metab (Seoul). 2020;35:416–24. 32615726 10.3803/EnM.2020.35.2.416 PMC7386102

[22] Qu P Mi Y Zhao D. Effect of the interaction between pre-pregnancy body mass index and fresh/frozen embryo transfer on perinatal outcomes of assisted reproductive technology-conceived singletons: a retrospective cohort study. Front Endocrinol. 2020;11:560103. 10.3389/fendo.2020.560103 PMC7546789 33101197

[23] Chen T-P Lin W-Y Chiang C-H. Metabolically healthy obesity and risk of non-alcoholic fatty liver disease severity independent of visceral fat. J Gastroen Hepatol. 2021;36:2903–10. 10.1111/jgh.15544 33973273

[24] Hong HC Lee J-S Choi HY. Liver enzymes and vitamin D levels in metabolically healthy but obese individuals: Korean national health and nutrition examination survey. Metabolism. 2013;62:1305–12. 23643404 10.1016/j.metabol.2013.04.002

[25] Wu L-M He H Chen G. Associations between obesity and metabolic health with nonalcoholic fatty liver disease in elderly Chinese. Hepatobiliary Pancreat Dis Int. 2020;19:252–7. 32205038 10.1016/j.hbpd.2020.02.010

[26] Man T Nolte IM Jaju D. Heritability and genetic correlations of obesity indices with ambulatory and office beat-to-beat blood pressure in the Oman Family Study. J Hypertens. 2020;38:1474–80. 32195815 10.1097/HJH.0000000000002430 PMC7374053

[27] Fan JG Wei L Zhuang H. Guidelines of prevention and treatment for non-alcoholic fatty liver disease: a 2018 update [In Chinese]. Zhonghua Gan Zang Bing Za Zhi. 2018;26:195–203. 29804393 10.3760/cma.j.issn.1007-3418.2018.03.008 PMC12769340

[28] Maltais-Payette I Boulet MM Prehn C. Circulating glutamate concentration as a biomarker of visceral obesity and associated metabolic alterations. Nutr Metab (Lond). 2018;15:78. 30450120 10.1186/s12986-018-0316-5 PMC6219091

[29] Perrin R Evans PM Webb S. The use of PET images for radiotherapy treatment planning: an error analysis using radiobiological endpoints. Med Phys. 2010;37:516–31. 20229860 10.1118/1.3276776

[30] Sung KC Seo DC Lee SJ. Non alcoholic fatty liver disease and risk of incident diabetes in subjects who are not obese. Nutr Metab Cardiovasc Dis. 2019;29:489–95. 30940491 10.1016/j.numecd.2019.01.016

[31] Du P Wang HJ Zhang B. Prevalence of abdominal obesity among Chinese adults in 2011. J Epidemiol. 2017;27:282–6. 28427831 10.1016/j.je.2017.03.001 PMC5463021

[32] Cuthbertson DJ Brown E Koskinen J. Longitudinal analysis of risk of non-alcoholic fatty liver disease in adulthood. Liver Int. 2019;39:1147–54. 30347485 10.1111/liv.13993

[33] Kim Y Han E Lee JS. Cardiovascular risk is elevated in lean subjects with nonalcoholic fatty liver disease. Gut Liver. 2022;16:290–9. 34238770 10.5009/gnl210084 PMC8924809

[34] Trifan A Rotaru A Stafie R. Clinical and laboratory characteristics of normal weight and obese individuals with non-alcoholic fatty liver disease. Diagnostics (Basel, Switzerland). 2022;12:801. 35453849 10.3390/diagnostics12040801 PMC9028454

[35] Hadi A Pourmasoumi M Najafgholizadeh A. The effect of apple cider vinegar on lipid profiles and glycemic parameters: a systematic review and meta-analysis of randomized clinical trials. BMC Complement Med Ther. 2021;21:179. 34187442 10.1186/s12906-021-03351-w PMC8243436

[36] Muzurović E Mikhailidis DP Mantzoros C. Non-alcoholic fatty liver disease, insulin resistance, metabolic syndrome and their association with vascular risk. Metabolism. 2021;119:154770. 33864798 10.1016/j.metabol.2021.154770

[37] Tan EX Lee JW Jumat NH. Non-obese non-alcoholic fatty liver disease (NAFLD) in Asia: an international registry study. Metabolism. 2022;126:154911. 34648769 10.1016/j.metabol.2021.154911

[38] Deng J Zhang Y Bu L. The prevalence, popular trends, and associated and predictive factors of non-obese fatty liver disease. Front Endocrinol. 2021;12:744710. 10.3389/fendo.2021.744710 PMC8485750 34603215

[39] Fox CS Massaro JM Hoffmann U. Abdominal visceral and subcutaneous adipose tissue compartments: association with metabolic risk factors in the Framingham heart study. Circulation. 2007;116:39–48. 17576866 10.1161/CIRCULATIONAHA.106.675355

[40] Eshraghian A Nikeghbalian S Geramizadeh B. Characterization of biopsy proven non-alcoholic fatty liver disease in healthy non-obese and lean population of living liver donors: the impact of uric acid. Clin Res Hepatol Gastroenterol. 2020;44:572–8. 31611031 10.1016/j.clinre.2019.09.002

[41] Wong VW Wong GL Chan RS. Beneficial effects of lifestyle intervention in non-obese patients with non-alcoholic fatty liver disease. J Hepatol. 2018;69:1349–56. 30142427 10.1016/j.jhep.2018.08.011

